# Is CO_2_ laser microsurgery better than radiotherapy in early glottic cancer: a meta-analysis

**DOI:** 10.1007/s10103-023-03890-3

**Published:** 2023-09-27

**Authors:** Yong Yang, Yong-li Wang, Li-zhi Wei, Ju-xin Wang, Fang-ting Huang, Guang-wu Huang

**Affiliations:** 1 Department of Otolaryngology-Head and Neck Surgery, The First Affiliated Hospital of Guangxi Medical University, No.6 of Shuangyong Road, Nanning, 530021 Guangxi China; 2 Department of Otolaryngology-Head and Neck Surgery, The First People’s Hospital of Nanning, Nanning, 530021 Guangxi China

**Keywords:** Early glottic cancer, Quality of life, Voice assessment/measures, Outcomes

## Abstract

The choice between radiotherapy (RT) and CO_2_ laser surgery (CO_2_-LS) for early glottic cancer remains controversial. We systematically examined electronic databases in order to identify prospective trials comparing patients who had undergone CO_2_-LS or RT to treat early glottic cancer. Eleven studies involving 1053 patients were included. In the selected literature, the parameter setting of CO_2_ laser equipment can be summarized as wavelength 10.6 µm, superpulsed mode, continuous setting, power tailored on target structures (1–3 W for subtle resections and 4–15 W for cutting a larger tumor), and approximately 2080–3900 W/cm^2^ of laser energy. Using RevMan 5.3, we estimated pooled odds ratios (ORs) for dichotomous variables and pooled mean differences (MDs) for continuous variables, along with associated 95% confidence intervals (CIs). The heterogeneity in the treatment variables was measured using Higgins’ inconsistency test and expressed as *I*^*2*^ values. The continuous variables were then depicted as histograms developed using PlotDigitizer 2.6.8. Compared to patients treated with CO_2_-LS, those treated with RT had better jitter (MD 1.27%, 95% CI 1.21 ~ 1.32, *P* < 0.001), and high scores on the “Grade (MD 6.54, 95% CI 5.31 ~ 7.76, *P* < 0.001), Breathiness (MD 9.08, 95% CI 4.02 ~ 14.13, *P* < 0.001), Asthenia (MD 2.13, 95% CI 0.29 ~ 3.98, *P* = 0.02), and Strain (MD 3.32, 95% CI 0.57 ~ 6.07, *P* = 0.02)” scale. Patients treated with CO_2_-LS had worse local control rates (OR 3.14, 95% CI 1.52 ~ 6.48, *P* = 0.002) while lower incidence of second primary tumor (OR 0.30, 95% CI 0.15 ~ 0.61, *P* < 0.001). It is hoped that retrospective analysis can provide suggestions for early glottis patients to choose personalized treatment.

## Introduction

The primary treatment for early glottic carcinoma (T1-T2) is RT or surgery. RT can achieve local control of the tumor, improve overall survival, and preserve breathing, airway integrity, and phonation [1, 2]. However, non-standardized RT can lead to recurrence in 5–35% of patients with early glottic carcinoma; this recurrence occurred mainly when the pretreatment tumor lay near dense lymph nodes and nerve tissue [3]. Patients who experience recurrence are also treated using transoral CO_2_-LS of the larynx, endolaryngeal cordectomy with cold instruments, or open partial laryngectomy. Although patients who undergo open partial laryngectomy experience good oncological results, this surgery is expensive, it can reduce voice quality, and it is associated with higher risk of postoperative complications [4, 5].

More recently, early glottic carcinoma has been treated using either CO_2_-LS or RT, since both these treatments are associated with good oncological and survival outcomes [6]. However, many patients may prefer CO_2_-LS over RT due to its association with minimal morbidity and high rate of laryngeal preservation, radiotherapy as the further adjuvant therapy after surgery after a [7, 8]. Furthermore, CO_2_-LS is considered to be a salvage treatment for recurrent laryngeal cancer after RT failure, since it can decrease the length of treatment and hospital stay, as well as reduce the incidence of side effects [9–11]. Nevertheless, the ongoing debate about the superior treatment for early glottic cancer continues, especially due to the lack of clinical data from prospective randomized trials.

A meta-analysis involving retrospective studies reported no significant differences between CO_2_-LS and RT with respect to local control of the tumor or overall survival [12]. Another study reported that transoral CO_2_-LS is an effective treatment for recurrent laryngeal cancer, and that is associated with high overall survival and high rates of local tumor control and larynx preservation [1]. Second meta-analysis showed that RT may be associated with longer maximum phonation time and lower fundamental frequency (f0) than laser surgery in the treatment of T1a glottic carcinoma [13].

In order to gain a better understanding of the efficacy of these two treatments, we performed a systematic review and meta-analysis of prospective trials and compared the voice quality and oncological outcomes of patients with early glottic carcinoma who underwent CO_2_-LS or RT. Here we assumed that CO_2_-LS is superior to RT on both voice quality and oncological outcomes.

## Materials and methods

### Search strategy

We systematically examined electronic databases, including Medline and PubMed (from 1946), Embase and OvidSP (from 1974), and the Cochrane Central Register of Controlled Trials (from 1965) in order to identify prospective trials comparing patients who had undergone CO_2_-LS or RT to treat early glottic cancer. The following search terms were used in combination with Boolean operators (AND or OR): “glottic cancer[All Fields],” “glottic carcinoma[All Fields],” glottic tumor[All Fields],” “vocal cord cancer[All Fields],” “vocal cord carcinoma[All Fields],” “vocal cord tumor[All Fields],” “laryngeal neoplasms[All Fields],” “larynx cancer[All Fields],” “larynx carcinoma[All Fields],” “larynx tumor[All Fields],” “laryngeal cancer[All Fields],” “laryngeal carcinoma[All Fields],” “laryngeal tumor[All Fields],” “transoral laser surgery[All Fields],” “transoral laser microsurgery[All Fields],” “transoral CO_2_ laser microsurgery[All Fields],” “CO_2_ laser cordectomy[All Fields],” “CO_2_ transoral microsurgery[All Fields],” “CO_2_ laser-assisted endoscopic surgery[All Fields],” “endoscopic resection[All Fields],” “surgery[All Fields],” “laser surgery[All Fields],” “gas lasers[All Fields],” “laser therapy[All Fields],” and “cordectomy[All Fields].” Only studies published in English before April 27, 2021, were considered. There were no restrictions based on year of publication or country of origin. All references cited in eligible publications were screened in order to ensure that relevant studies were not overlooked.

### Study selection

After systematically examining the records indexed in the electronic databases, we identified studies comparing the efficacy of using CO_2_-LS and RT, or a combination of the two, to treat patients with early-stage glottic carcinoma according to the criteria in the original references. After excluding duplicate records, we reviewed the titles and abstracts, followed by the full text to identify prospective comparative studies for our meta-analysis. We included only studies involving patients with a confirmed diagnosis of early glottic carcinoma. The retrospective, ongoing or single-arm studies were excluded.

### Data extraction

Three authors (Y. Y., Y.-L. W., and J.-X. W.) independently extracted data from included studies according to the Preferred Reporting Items for Systematic Reviews and Meta-Analyses. Y. Y., a Ph.D. student, has been working in surgical treatment of head and neck sub-specialty for 18 years. He is well-experienced in the diagnosis and treatment of early pharyngeal cancer and CO_2_-LS of early vocal cord cancer. Y.-L. W., an M.D., has been working in clinical otolaryngology for 14 years. He engages in observing and evaluating voice after head and neck tumor radiation therapy, as well as collecting and analyzing clinical data. J.-X. W. has been working in clinical otolaryngology for 8 years. He is responsible for the follow-up of discharged patients and the speech function recovery of postoperative patients. We extracted data on voice quality, oncologic outcomes, and quality of life from each included study. For the purpose of this meta-analysis, we considered all patients who had undergone both CO_2_-LS as the intervention group, and those who had undergone RT as the control group.

In the efficacy comparisons, the primary outcome was voice quality, which included the following variables: jitter, shimmer, f0, noise/harmonic ratio, maximum phonation time, normalized noise energy, and scores on the “Grade, Roughness, Breathiness, Asthenia, and Strain” (GRBAS) scale [14]. The secondary outcomes included self-assessment of voice quality and voice-related quality of life scores, as well as oncological outcomes such as incidence of local tumor control, recurrence, second primary tumors, and death.

### Selection criteria

Epidemiological studies comparing CO_2_-LS to RT in patients with early-stage glottic carcinoma were performed. After excluding all duplicated studies, prospective comparative studies were selected from the reading of titles and abstracts. When it was not definite whether the study would be included, the full text was screened for more detailed analysis. Studies include patients with a confirmed diagnosis of early glottic carcinoma. The intervention group was considered the patients submitted to CO_2_-LS and RT. The control group was considered the patients that were submitted to radiotherapy.

### Data extraction

Three authors (Y. Y., Y.-L. W., and J.-X. W.) used a predefined protocol to independently identify the studies. For comparing patients undergoing CO_2_-LS or RT, the primary outcome was voice quality including following variables: Jitter, Shimmer, f0, noise/harmonic ratio (NHR), maximum phonation time (MPT), normalized noise energy (NNE), and GRBAS scores with Grade, Roughness, Breathiness, Asthenia, and Strain. The second outcomes included self-assessment of voice quality (VHI), voice-related quality of life (VRQOL) score, and the oncological outcomes such as incidence of local control, recurrence, death, and second primary tumors.

### Quality assessment

The quality of the methodology used in the included studies was evaluated using RevMan 5.3 (RevMan; The Cochrane Collaboration, Oxford, UK). The risk of bias in the randomized trials was independently assessed by two authors (L.-Z. W. and F.-T. H.) based on previously described methods [15], which took into account random sequence generation, intervention allocation, blinding of participants, assessments of outcomes (including incomplete outcomes), and selective reporting in all included studies. L.-Z. W. has been working in clinical otolaryngology for more than 20 years. He is expert in postoperative voice function recovery work. F.-T. H has been working in clinical otolaryngology for over 5 years and is in charge of the follow-up of postoperative patients.

### Power analysis

G*Power software (latest ver. 3.1.9.7; Heinrich-Heine-Universität Düsseldorf, Düsseldorf, Germany) is a free statistical software specifically designed for calculating statistical power (including sample size). In our study, G*Power software was used to calculate the statistical power of meta-analysis. For each study, the null hypothesis of equal positive proportions in two populations was tested, with a significance level (α) set as 0.05. This two-tailed test made sure that the impact can be interpreted in either direction. The statistical power of each study was calculated based on the number of proposed study groups.

### Statistical analysis

Using RevMan 5.3, we estimated pooled odds ratios (ORs) for dichotomous variables and pooled mean differences (MDs) for continuous variables, along with associated 95% confidence intervals (CIs). The heterogeneity in the treatment variables was measured using Higgins’ inconsistency test and expressed as *I*^*2*^ values: An *I*^*2*^ value of 25% indicated low heterogeneity, 50% indicated moderate heterogeneity, and 75% indicated high heterogeneity. We used the inverse-variance fixed effects model for outcomes showing low heterogeneity (< 25%), and the DerSimonian and Laird random effects model for outcomes showing moderate to high heterogeneity (> 50%). We estimated means and standard deviations for continuous variables that were published as medians and interquartile ranges using an online tool (http://www.comp.hkbu.edu.hk/~xwan/median2mean.html) and previously established methods [16, 17]. The continuous variables were then depicted as histograms developed using PlotDigitizer 2.6.8 (version of 27 October 2015, Sun Microsystems, Philippe Zeller, French). *P* < 0.05 was set as the level of significance.

## Results

### Search strategy

**Fig. 1:**
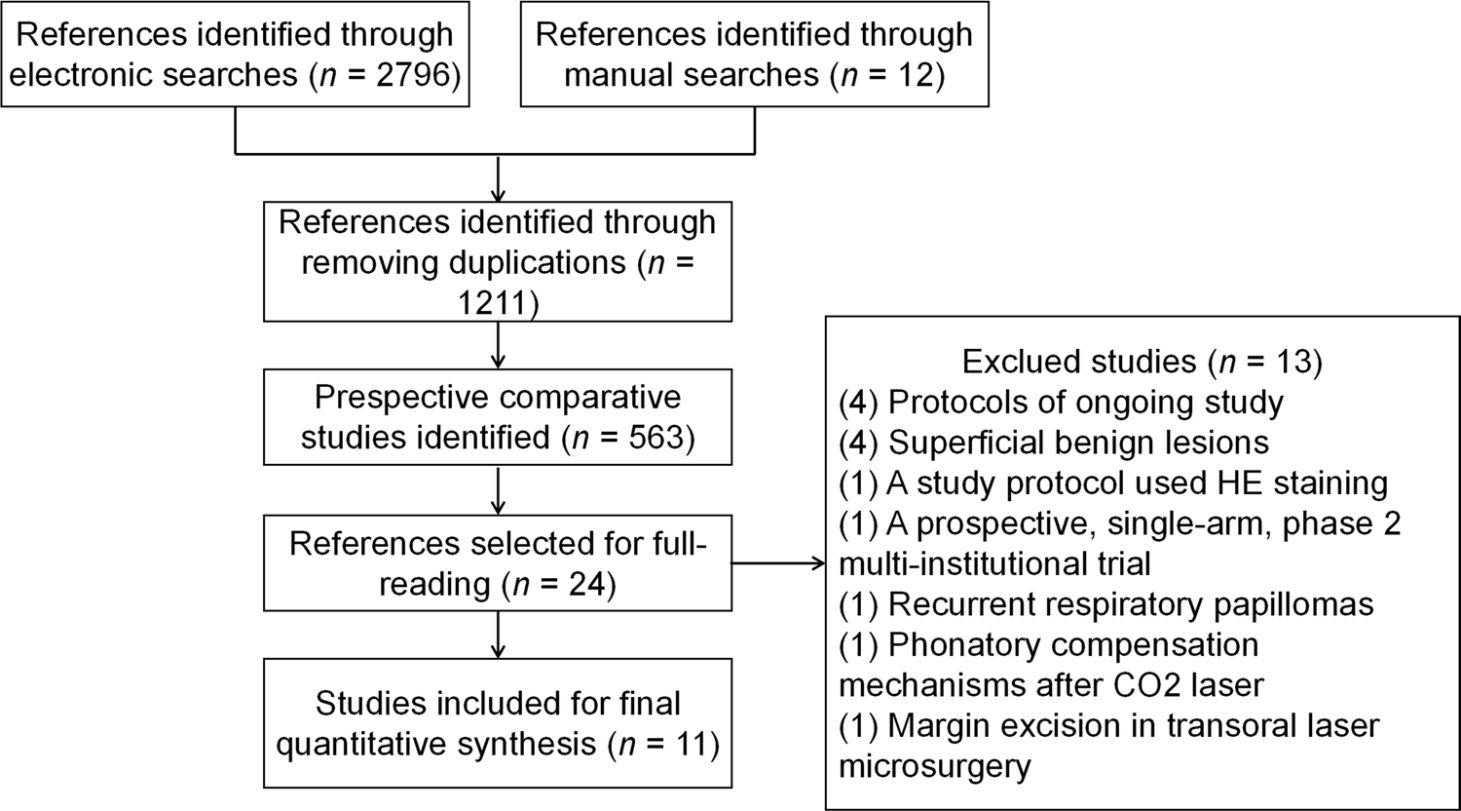
Flowchart of study selection

A total of 2806 studies were identified from the databases examined (Fig. 1). After removing duplicates and reviewing the titles and abstracts of the remaining studies, 23 prospective comparative studies were retained for the full-text analysis. After considering the eligibility criteria, eleven unique studies involving 1053 patients were included in the final meta-analysis.

We excluded a total of 13 studies: four ongoing protocols, four studies involving patients with superficial benign lesions, one study using hematoxylin–eosin staining, one prospective single-arm trail, one study involving patients with recurrent respiratory papillomas, one study where patients received interventions related to phonatory compensation mechanisms after CO_2_-LS, and one study where margin excisions were performed during transoral laser microsurgery (Fig. 1).

### Characteristics of included studies

Of the eleven studies included [11, 14, 18–26], nine studies [11, 19–26] were prospective comparative or cohort studies, while the other two were randomized, parallel-group studies [14, 18] (Table 1). The effect of CO_2_-LS treatment was compared to full-dose radiotherapy in four studies [19, 21, 23, 26], to 2 Gy/d radiotherapy in two other studies [14, 22], and to 225 cGy/session radiotherapy in two other studies [11, 24]. The other studies [18, 20, 25] compared CO_2_-LS treatment with 6-MV or ^60^Co photon treatment, or to 225 cGy/d and 2.5 Gy/fraction radiotherapy. Patient follow-up was > 24 months in all included studies.

**Table 1:** General introduction of included studies

Study	Design	Years	Sample	Tumors	Demography	Treatment (*n*)	Outcomes	Follow-up
Rydell [26]	Prospective comparative study	1982–1991	36	T1a glottic squamous cell carcinoma	Mean age 65.2 vs 65.1; Both male	CO_2_ laser cordectomy (18) Full dose radiotherapy (18)	Quality of voice including breathiness, Jitter, F0 average, sample text reading time and number of breaths	24 months
Krengli [25]	Prospective comparative study	1990–2001	36	T1a glottic carcinoma	Mean age 67.5 vs 69; 55 male and 2 female	CO_2_ laser cordectomy (20) 6MV or ^60^Co photons radiotherapy (16)	Quality of voice including Yanagihara, Jitter, Shimmer, NHR and F0 average; Local control, recurrence, salvage therapy, diplophonia	24–120 months
Núñez [24]	Prospective comparative study	NM	37	T1 glottic carcinoma	Mean age 64 vs 67; Both male	CO_2_ laser surgery (19) 225 cGy/d radiotherapy (18)	Quality of voice included Grade class, GRBAS, voice handicap index, Jitter, Shimmer, F0 average, MPT and NNE	30 months vs 43 months
Oridate [23]	Prospective comparative study	2006–2007	77	T1 glottic carcinoma	Mean age 79 vs 71; Both male	CO_2_ laser surgery (14) Radiotherapy (63)	GRBAS, VRQOL and VHI-10 scores	24 months vs 6 months
Mahler [22]	Prospective comparative study	1986–2005	351	T1a glottic carcinoma	NM	CO_2_ laser surgery (188) 2 Gy/d radiotherapy (163)	Local control, recurrence, laryngectomy, second primary tumors, disease-specific survival, estimated 5-year survival	29 months
Van [20]	Prospective cohort study	NM	106	T1a glottic carcinoma	Mean age 66 vs 66; Both male	CO_2_ laser surgery (67) 2.5 Gy/fraction radiotherapy (39)	Quality of voice included Jitter, Shimmer, NNE and F0 average Recurrence, laryngectomy,	24 months
Aaltonen [14]	Randomized, parallel-group study	1998–2008	60	T1a glottic squamous cell carcinoma	Mean age 69 vs 61; Both male	CO_2_ laser surgery (32) 2 Gy/d radiotherapy (28)	Quality of voice included GRBAS, breathiness and asthenia, hoarseness and impact of everyday life Glottal function, VAS	24 months
Zhang [18]	Single-Blind Randomized Clinical Trial	2010–2014	123	T1a glottic cancer 53,880.57	Mean age 56.71 vs 57.15; 108 male and 23 female	CO_2_ laser surgery (62) LTP-RFA (61)	Three year overall survival rate, glottal function, Quality of voice included Grade class, roughness, breathiness, asthenia, strain, Jitter, Shimmer, F0 average, MPT and NHR	33 months vs 36 months

*NHR*, noise/harmonic ratio; *GRBAS*, perceptual dysphonia analysis; *MPT*, maximum phonation time; *F0 (Hz)*, fundamental frequency; *NNE*, normalized noise energy; *VHI-10*, Voice Handicap Index-10; *VRQOL*, Voice-Related Quality of Life; *NM*, not mentioned; *LTP-RFA*, low-temperature plasma radiofrequency ablation

### Quality assessment

**Fig. 2:**
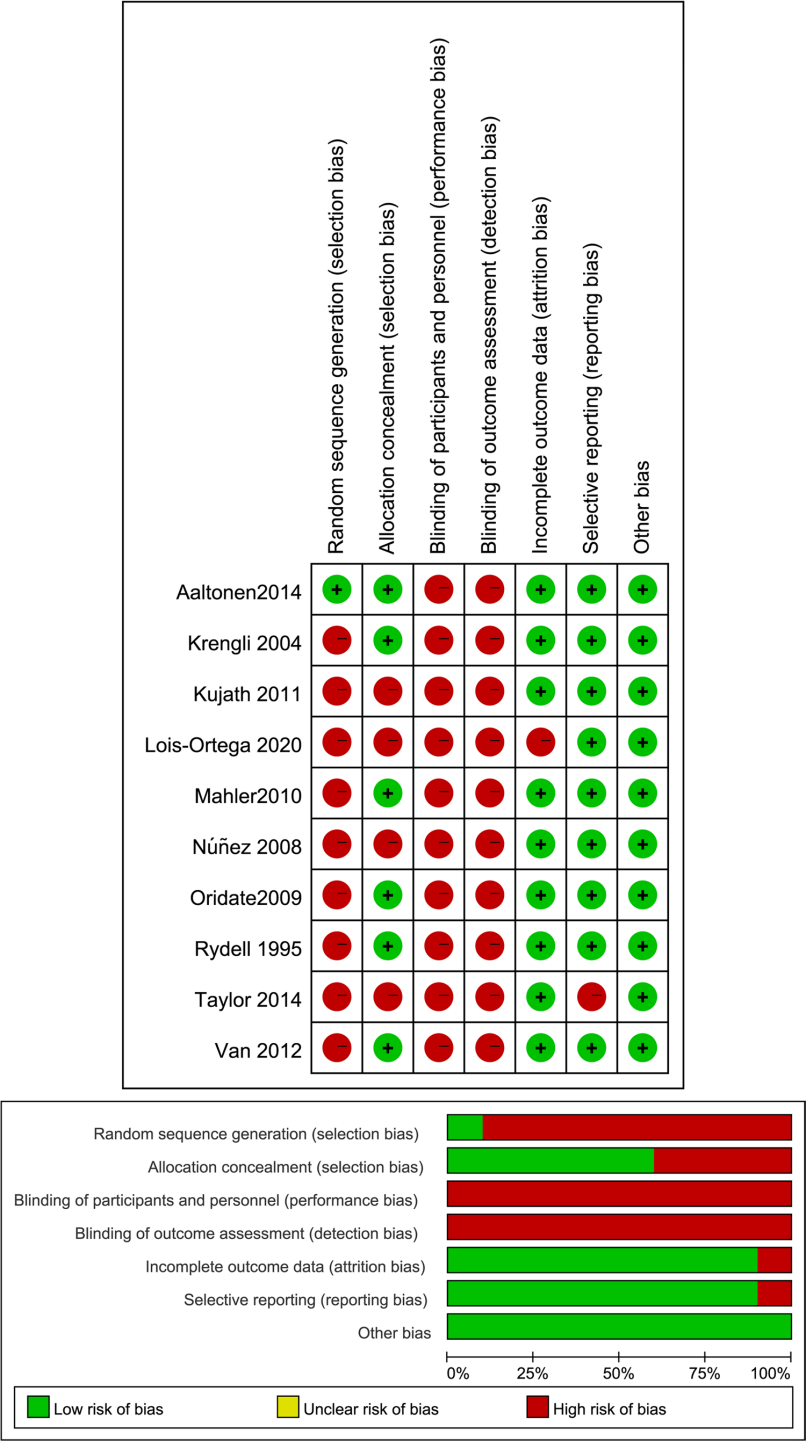
Risk of bias assessment in included studies. **A** Risk of bias graph: review authors’ judgements about each risk of bias item presented as percentages across all included studies. **B** Risk of bias summary: review authors’ judgements about each risk of bias item for each included study

Only one study [14] described the method of random sequence generation with low selection bias. Six studies [14, 20, 22, 23, 25, 26] reported the method used for allocation concealment. Unfortunately, there were no studies demonstrated how they blinded the participants and the outcome assessments. Almost all included studies, except one, showed the high risk of attrition [11] and reporting bias [19], respectively. Other bias was considered low in all included studies (Fig. 2).

### Voice quality assessment

**Fig. 3:**
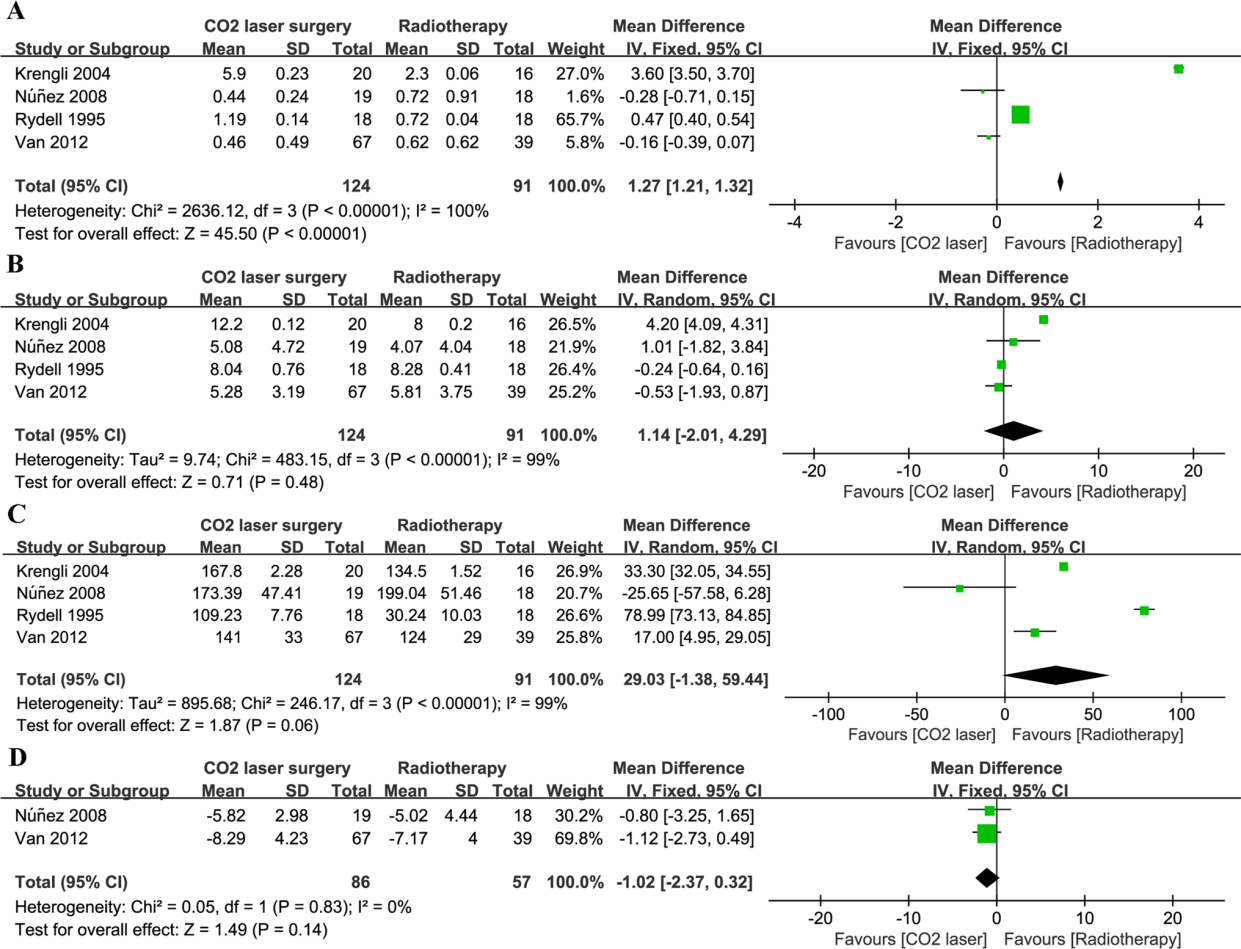
Pooled risk for voice quality with CO_2_-LS versus RT. **A** Jitter score; **B** Shimmer score; **C** F (0); **D** NNE. Abbreviations: SD, standard deviation; IV, inverse-variance; CI, confidence interval

**Fig. 4:**
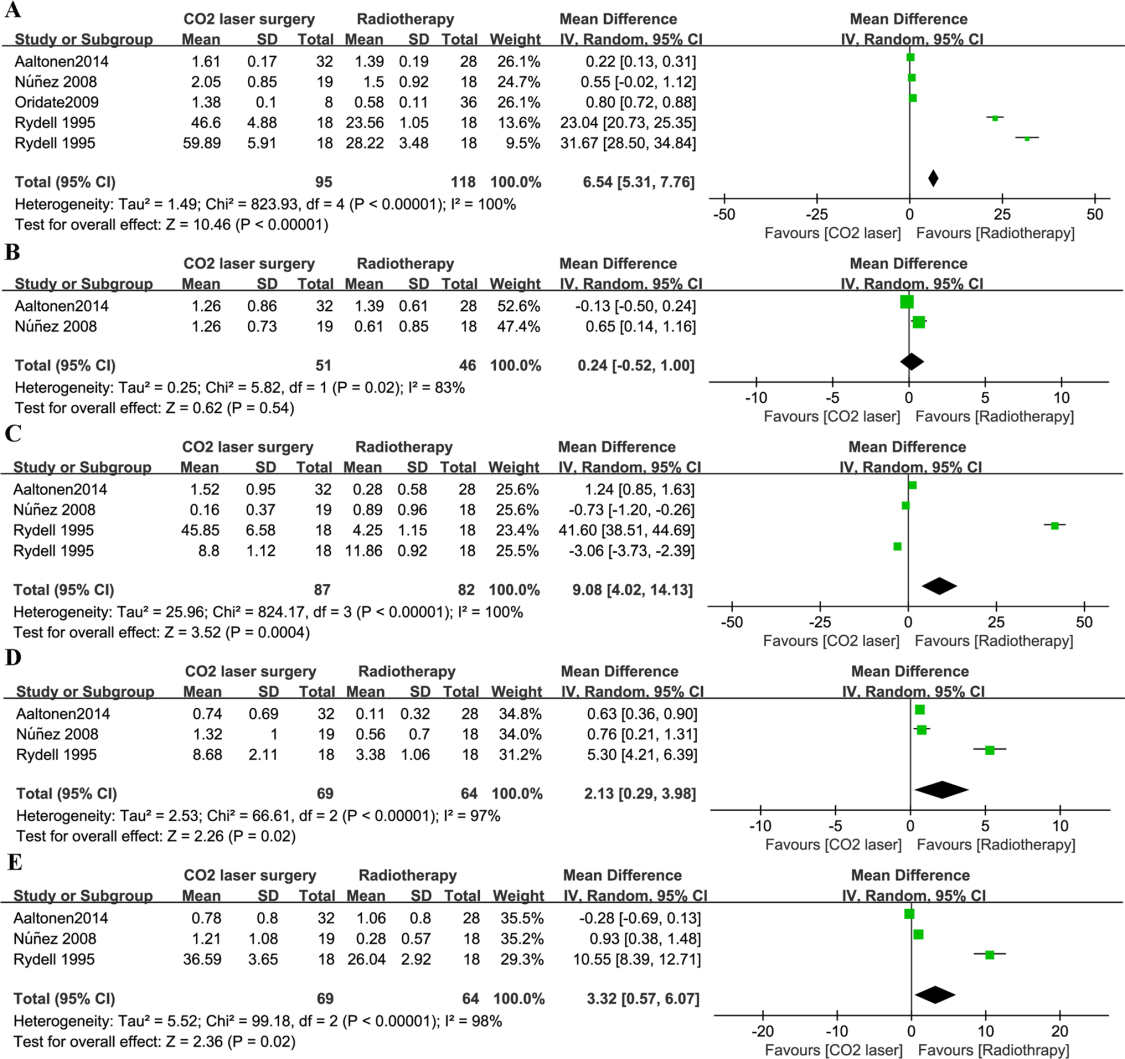
Pooled risk for GRBAS with CO_2_-LS versus RT. **A** Grade score; **B** Roughness score; **C** Breathiness score; **D** Asthenia score; **E** Strain score. Abbreviations: SD, standard deviation; IV, inverse-variance; CI, confidence interval

A pooled analysis based on data from four studies [20, 24–26] showed that patients who had undergone RT had lower jitter scores (MD 1.27, 95% CI 1.21 to 1.32, *P* < 0.001; Fig. 3A) than those who had undergone CO_2_-LS. There were no significant differences in shimmer score (Fig. 3B), f0 values (Fig. 3C) based on data from four studies [20, 24–26], or NNE (Fig. 3D) based on data from two studies [20, 24]. There was only one study that reported the results of NHR [25] and MPT [24], respectively, and found that patients treated with RT showed lower NHR and MPT compared to those treated with CO_2_-LS. The GRBAS scale was used to conduct an auditory-perceptual evaluation. A total of four studies [14, 23, 24, 26] reported that RT was associated with significantly higher *Grade* scores than CO_2_-LS (MD 6.54, 95% CI 5.31 to 7.76, *P* < 0.001; Fig. 4A). Similarly, RT was associated with high scores for *Breathiness* (3 studies, 169 patients; MD 9.08, 95% CI 4.02 to 14.13, *P* < 0.001; Fig. 4C), *Asthenia* (3 studies, 133 patients; MD 2.13, 95% CI 0.29 to 3.98, *P* = 0.02; Fig. 4D), and *Strain* (3 studies, 133 patients; MD 3.32, 95% CI 0.57 to 6.07, *P* = 0.02; Fig. 4E). However, there were no significant differences in *roughness* (Fig. 4B) based on data from two studies [14, 24].

### Life quality assessment

Only one study [23] included in this meta-analysis reported results related to self-assessment of voice quality and voice-related quality of life scores. Another study [21] found that CO_2_-LS resulted in less likely to be understood all the time (Performance Status Scale for Head and Neck Cancer Patients: understandability score 100; OR = 12.2; *P* = 0.03) and a higher likelihood of having a VHI-10 score of 10 or more (OR = 16.2; *P* = 0.001). Nevertheless, Taylor et al. [19] also reported that VHI-10 ranged from 0 to 11 (median 6) in the CO_2_-LS group and 0 to 34 (median 7) in the RT group during the last work up. Hence, patients treated with RT reported better self-assessment of voice quality, while those treated with CO_2_-LS reported better voice-related quality of life.

### Oncological outcomes

**Fig. 5:**
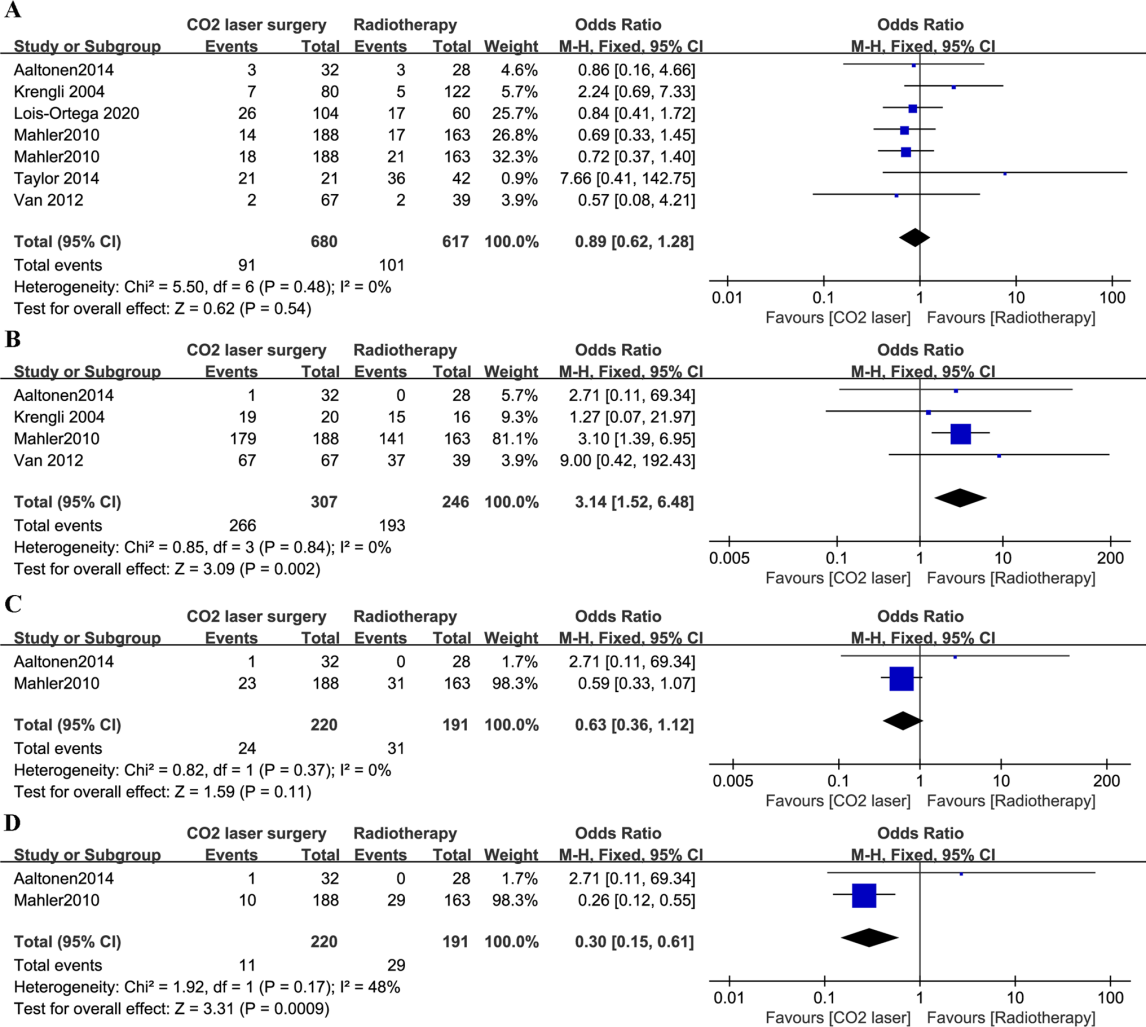
Pooled risk for oncological outcomes with CO_2_-LS versus RT. **A** Recurrence; **B** local control; **C** death; **D** second primary tumors. Abbreviations: M-H, Mantel–Haenszel; CI, confidence interval

Based on data from six studies [11, 14, 19, 20, 22, 25], there were no significant differences in recurrence rates between patients treated with CO_2_-LS or RT (*P* = 0.54; Fig. 5A). Pooled analysis showed that patients treated with RT had lower incidence of local tumor control (MD 3.14, 95% CI 1.52 to 6.48, *P* = 0.002; Fig. 5B) but increased the risk of second primary tumors (MD 0.30, 95% CI 0.15 to 0.61, *P* < 0.001; Fig. 5D) than those treated with CO_2_-LS. No statistical differences were observed between the two groups in the incidence of death (*P* = 0.11; Fig. 5C).

### Power analysis

As shown in Table 2, there were the results of pooled risk for voice quality when CO_2_-LS versus RT. In the meta-analysis of jitter scores, 3 of 4 studies [24–26] achieved 80% power. As for the shipper score, only 1 of 4 studies [26] achieved 80% power. In the meta-analysis of f0 values, 2 out of 4 studies [25, 26] achieved 80% power. The power of 2 studies [20, 24] in NNE’s meta-analysis did not exceed 80%.

**Table 2:** Power analysis of the studies (pooled risk for voice quality with CO_2_-LS versus RT)

Study or subgroup	jitter scores	shimmer score	f0 values	NNE
Rydell [26]	1.000	1.000	1.000	/
Krengli [25]	1.000	*	1.000	/
Núñez [24]	0.932	0.097	0.360	0.125
Van [20]	0.362	0.129	0.717	0.256

“/” is not included in this meta-analysis for this study; “*” represents effect size d is outside the acceptable range − 20–20

The results of pooled risk for GRBAS when CO_2_-LS versus RT are shown in Table 3. In the meta-analysis of Grade scores, 5 out of 6 studies [14, 24, 26, 27 (1) and (2)] achieved 80% efficacy. As for Roughness, the power of 2 studies [14, 24] did not reach 80%. In the meta-analysis of scores for Breathiness, 4 studies [14, 25, 27 (1) and (2)] all achieved 80% power. In Asthenia’s meta-analysis, 2 out of 3 studies [14, 26] achieved 80% power. Only 1 out of 3 studies in the meta-analysis of Strong [26] achieved 80% power.

**Table 3:** Power analysis of the studies (pooled risk for GRBAS with CO_2_-LS versus RT)

Study or subgroup	Grade scores	Roughness	Breathiness	Asthenia	Strain
Rydell [26] (1)	1.000	/	1.000	1.000	1.000
Rydell [26] (2)	1.000	/	1.000	/	/
Krengli (2004)	1.000	/	/	/	/
Núñez (2008)	0.481	0.749	1.000	0.613	0.721
Oridate (2009)	1.000	/	/	/	/
Aaltonen (2014)	0.998	0.089	0.999	0.934	0.265

“/” is not included in this meta-analysis for this study

## Discussion

In this meta-analysis, we aimed to compare the efficacy of CO_2_-LS or RT for treating patients with early glottic carcinoma. The collected data showed that patients treated with CO_2_-LS had a better quality of life and were able to achieve local control of the tumor than those treated with RT. However, patients treated with RT had better overall voice quality, reflected in lower jitter, shimmer, and f0, as well as lower noise/harmonic ratios and GRBAS scores. These findings indicate that CO_2_-LS is associated with better local control rates and quality of life for patients with early glottic cancer.

Although total laryngectomy was previously considered the primary treatment for recurrent laryngeal cancer after RT [22, 23], it is associated with high incidence of postoperative complications, so more radical and invasive procedures are needed. CO_2_-LS has many advantages over total laryngectomy, including precise cutting, bloodless operation, short operation time and hospital stay, reduced cost of hospitalization, and decreased recurrence rates [24–26]. Therefore, CO_2_-LS has recently begun to be used as a salvage surgery for patients with recurrent early glottic carcinoma. A treatment goal for patients with early glottic cancer is maintaining and improving voice quality. Some studies reported no differences in voice quality after RT or CO_2_-LS, while others reported better voice quality after RT [13, 27, 28]. RT can allow more precise, local treatment at the tumor location and reduce damage to normal cells, which is crucial for maintaining normal glottis function [29–31]. This may help explain the higher voice quality after RT than after CO_2_-LS in our study, and also reflects the one-sidedness of the assumptions in the “Introduction” section. On the other hand, patients in our analysis showed higher voice-related quality of life scores after CO_2_-LS than after RT. This may reflect that the procedure was associated with shorter operation time, shorter hospital stay, lower hospitalization costs, and lower recurrence rates than RT.

Oncological results are the main outcomes used to evaluate the treatment of cancer. In a retrospective study [32], revision transoral LS is confirmed the oncological radicality in most cases, even in the case of positive, close, or non-evaluable margins. One study reported high 5-year rates of 75–84% for local tumor control, overall laryngeal preservation, overall survival, and disease-specific survival after CO_2_-LS [33]. Other studies showed similar rates of local tumor control with CO_2_-LS or RT [34, 35]. The pooled results of prospective studies in the present meta-analysis showed that CO_2_-LS was associated with better local control rates than RT, but the two techniques were associated with similar rates of recurrence, death, and second primary tumors.

As far as we know, this is the first meta-analysis to include prospective comparative trials and exclude all retrospective studies. Furthermore, we objectively analyzed oncologic outcomes, as well as voice and life quality after treatment with CO_2_-LS and RT in a total of 826 participants. Our findings suggest that both CO_2_-LS and RT are excellent options for the treatment of patients with early glottic cancer. Although patients treated with CO_2_-LS may have better local control rates and quality of life, those treated with RT may have superior voice quality. Therefore, clinicians should consider each patient’s situation before recommending treatment with CO_2_-LS or RT. Further large, high-quality, double-blind, prospective controlled trials are needed in order to gain a better understanding of the efficacy of both treatments.

Meta-analysis is a statistical method that combines multiple homogeneous studies on the same topic using quantitative methods to obtain overall results. It can conduct a significance test to analyze whether the interventions and exposures are meaningful or not. Therefore, ensuring the statistical power of the study is crucial. We calculated the statistical efficiency of this meta-analysis. Since there were 1–2 studies with a statistical power less than 80% in each meta-analysis, it indicated that the statistical efficiency of meta-analysis in this study needed to be improved.

## Conclusion

Neither CO_2_-LS nor RT can solve all the problems in the treatment of patients with early glottic cancer patients. Although patients treated with CO_2_-LS had significant better local control rates and quality of life, those treated with RT should be associated with recovery of voice quality. Therefore, clinicians must consider the specific situation of the individual with early glottic cancer before recommending treatment with more comprehensive and cautious to apply CO_2_-LS or RT for early glottic cancer patients according to the specific situation of the individual.
